# Mental illness after bereavement before and during the COVID-19 pandemic in Sweden: A matched cohort study

**DOI:** 10.1371/journal.pmen.0000565

**Published:** 2026-05-06

**Authors:** Shiyu Li, Mary M. Barker, Huiqi Li, Krisztina D. László, Fen Yang, Mikael Rostila, Sandra Rogne, Filip K. Arnberg, Maria Feychting, Unnur A. Valdimarsdóttir, Fredrik Nyberg, Fang Fang

**Affiliations:** 1 Institute of Environmental Medicine, Karolinska Institutet, Stockholm, Sweden; 2 School of Public Health and Community Medicine, Institute of Medicine, Sahlgrenska Academy, University of Gothenburg, Gothenburg, Sweden; 3 Department of Global Public Health, Karolinska Institutet, Stockholm, Sweden; 4 Department of Public Health and Caring Sciences, Uppsala University, Uppsala, Sweden; 5 Department of Public Health Sciences, Stockholm University, Stockholm, Sweden; 6 Centre for Health Equity Studies (CHESS), Stockholm University/Karolinska Institutet, Stockholm, Sweden; 7 Centre for Health Equity Studies, Stockholm University/Karolinska Institutet, Stockholm University, Stockholm, Sweden; 8 Department of Medical Sciences, National Centre for Disaster Psychiatry, Uppsala University, Uppsala, Sweden; 9 Centre of Public Health Sciences, Faculty of Medicine, University of Iceland, Reykjavik, Iceland; 10 Department of Epidemiology, Harvard T H Chan School of Public Health, Boston, Massachusetts, United States of America; Baylor Scott and White Research Institute, UNITED STATES OF AMERICA

## Abstract

Bereavement is associated with an increased risk of mental illness. The COVID-19 pandemic caused excess mortality, and may have exacerbated the mental health impact of bereavement due to social restrictions and reduced healthcare access. Using Swedish national register data, this study aimed to compare the risk of mental illness following bereavement before (2018–2019) and during (2020–2021) the pandemic, exploring how the pandemic might have modified the psychological impact of bereavement and identifying high-risk groups.We conducted a nationwide matched cohort study including (1) 3,840,845 individuals (349,168 bereaved) before the pandemic, and (2) 5,132,988 individuals (466,636 bereaved) during the pandemic. Mental illness was defined as the first occurrence of any psychiatric diagnosis or suicidal behavior during each period. Multivariable Cox regression was used to estimate hazard ratios (HRs) with 95% confidence intervals (CIs). We found that bereaved individuals had a significantly higher risk of mental illness compared to non-bereaved individuals in both periods (before pandemic: HR 1.42, 95%CI 1.34-1.49; during pandemic: HR 1.34, 95%CI 1.28-1.39). Bereaved individuals younger than 30 years had markedly higher risks of psychiatric disorders in the pre-pandemic period compared to the pandemic period. Higher risks of incident psychiatric disorders were observed for loss of a child or spouse, compared to loss of a sibling or parent, as well as for loss due to accident or suicide as compared to other causes. Furthermore, bereavement due to COVID-19 was associated with an increased risk of mental illness during the pandemic period (HR 1.38, 95% CI 1.21-1.59). In conclusion, bereavement was consistently associated with an increased risk of mental illness, before and during COVID-19 pandemic, although young individuals (<30 years) seemed more affected before the pandemic. However, further research in settings with a different pandemic burden and/or mitigation strategies is needed to assess the generalizability of our findings beyond Sweden.

## Introduction

Bereavement is a profoundly stressful life event associated with a wide range of adverse health outcomes, including mental illness [1–4]. The experience of bereavement acts as a major stressor that can adversely affect an individual’s adaptive capacity through both the primary loss and secondary stressors, such as the loss of meaningful social roles and the disruption of daily life routines. [5]. Studies have shown that bereaved individuals have higher risks of depression, anxiety, substance use disorder, and suicidal behavior, compared to the general population [6–8]. Such risk varies by life stage and the nature of the loss. For example, loss of a parent during childhood, loss of a spouse in later life, and loss due to sudden deaths such as suicide are associated with particularly high risk [9–11].

The Coronavirus disease 2019 (COVID-19) pandemic has led to excess mortality [12]. As of January 2024, the virus had caused more than 27,000 COVID-deaths in Sweden [13], leaving approximately 240,000 individuals bereaved based on kin loss multipliers derived from U.S. data [14]. Pandemic-related stressors could have amplified the psychological impact of loss due to social isolation, inability to perform end-of-life rituals, restrictions on funeral gatherings, and reduced availability of mental health services [15]. For instance, some studies have suggested that loss due to COVID-19 and disrupted funeral rituals can intensify grief symptoms [16,17]. Nonetheless, existing studies have mostly relied on small-scale, cross-sectional and self-reported data, which is often limited by selection and information biases.

The distinctive mitigation strategies in Sweden, which avoided strict lockdowns in favor of voluntary measures [18–20], offers an opportunity to examine bereavement outcomes without compulsory home confinement. While research indicates a slight decline in specialist psychiatric consultations during the early phase of the pandemic in Sweden, potentially reflecting an overall reduction in healthcare utilization [21], it is unclear how such service disruptions affected bereaved individuals. Using nationwide high-quality register data in Sweden, we performed two cohort studies, identifying all bereaved individuals and ascertaining different types of mental illness across the entire population, both before and during the COVID-19 pandemic.

We hypothesized that the COVID-19 pandemic acted as an additional stressor, potentially exacerbating the mental health impact of bereavement. Leveraging Swedish nationwide register data, the present study aimed to compare the risk of incident mental illness following bereavement between the pre-pandemic (2018–2019) period and the pandemic (2020–2021) period.

## Methods

### Study design and participants

We conducted two nationwide matched cohort studies using data from the SCIFI-PEARL (Swedish COVID-19 Investigation for Future Insights—a Population Epidemiology Approach using Register Linkage) project. SCIFI-PEARL is a regularly updated, nationwide register-based study, including linked data from different population and health registers, including COVID-19 status, at individual level using the unique Swedish Personal Identity Number [22]. Participants were selected through the Swedish Total Population Register (TPR) and the Multi-Generation Register (MGR). The TPR includes information on different life events including birth, death, marital status change, family relationships, and migration within Sweden as well as to and from other countries [23], whereas the MGR includes largely complete information on familial links for all Swedish residents born since 1932 in Sweden [24]. Consequently, our ability to identify bereavement events was limited for older participants (e.g., participants aged 75+), as those with parents born before 1932 did not have linked parental data.

The SCIFI-PEARL project received pseudonymized data prepared by the National Board of Health and Welfare (NBHW) and Statistics Sweden, which are stored on a secure server at the Centre of Registers Västra Götaland [22]. Ethical approval was obtained from the Swedish Ethical Review Authority (2020–01800), which waived the requirement for informed consent because existing pseudonymized register data were used. Data access was granted only after the SCIFI-PEARL project team reviewed and approved the data extraction plan, ensuring that only variables necessary for this study were included. The research team accessed the pseudonymized data for research purposes remotely on 22/04/2024. All individual-level data remained on the secure server, and only aggregated results were allowed migrated outside.

To optimize the accuracy of individual-level information in Swedish national registers and to ensure equal maximum follow-up duration across periods, we defined the pre-pandemic period as January 1, 2018 - December 31, 2019, and the pandemic period as January 1, 2020 -December 31, 2021.

#### Study population and follow-up for study period 1 (pre-pandemic, Jan. 1^st^, 2018 - Dec. 31^st^, 2019).

**Fig 1:**
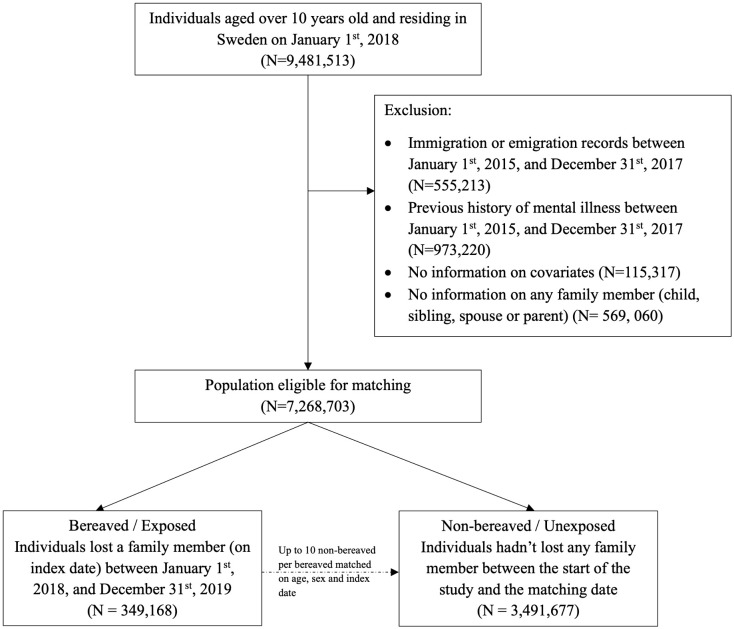
Flowchart of the matched cohort design for bereaved and their individually matched non-bereaved individuals in the pre-pandemic period (January 1^st^, 2018 – December 31^st^, 2019).

Individuals aged 10 or older, who were residing in Sweden and had at least one living family member (child, spouse, sibling, or parent) on December 31st, 2017 were included in the analysis of study period 1 (Fig 1). Individuals were excluded if they had a clinical diagnosis of any psychiatric disorder (ICD-10, F10-F99) or suicide attempt (ICD-10, X60-X84, Y10-Y34), [25,26], or immigration or emigration record between January 1st, 2015 (earliest date available) and December 31st, 2017, or if they had no valid information on age, sex, education, or household income. Among the remaining individuals, we first identified all individuals who experienced at least one loss of family member between January 1st, 2018, and December 31st, 2019 (i.e., exposed). The date of first loss was defined as the index date. We then used the method of exposure density sampling [27] to match the exposed individuals to up to 10 individuals who were unexposed at the index date, by birth year and sex. All participants were followed from the index date (i.e., start of the follow-up) until the first occurrence of mental illness, death, emigration, study period 1 end date (December 31^st^, 2019), or experience of loss (unexposed individuals only), whichever came first.

#### Study population and follow-up for study period 2 (during pandemic, Jan. 1^st^, 2020 - Dec. 31^st^, 2021).

**Fig 2:**
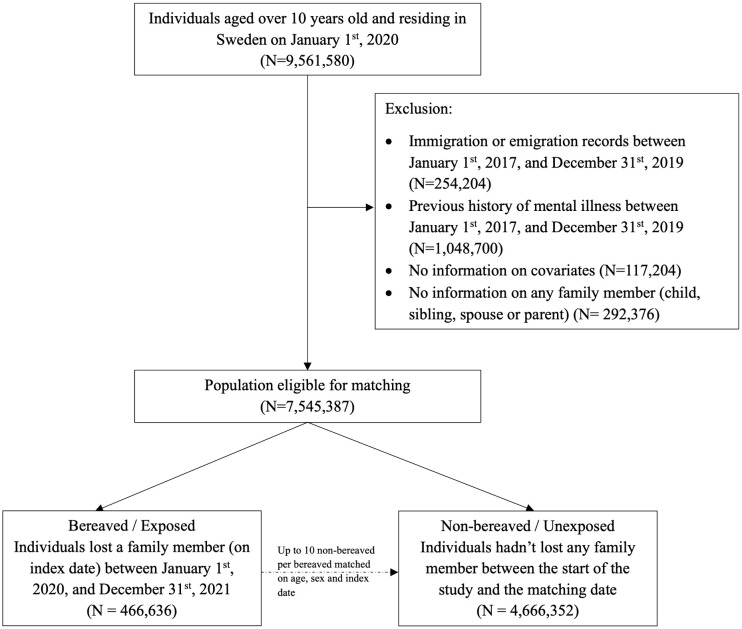
Flowchart of the matched cohort design for bereaved and their individually matched non-bereaved individuals in the pandemic period (January 1^st^, 2020 – December 31^st^, 2021).

Individuals aged 10 years or older, who were residing in Sweden and had information on at least one living family member on January 1st, 2020 were included in the analysis of study period 2 (Fig 2). Exclusion criteria and the selection of exposed and unexposed individuals were similar to that described in case of study period 1. Individuals included in the analysis for study period 1 who remained free of mental illness were eligible for inclusion in the analysis for study period 2 if they met the inclusion criteria on January 1st, 2020. The exposed and unexposed individuals were followed from the index date until the first occurrence of mental illness, death, emigration, end of study period 2 (December 31st, 2021), or experience of loss (unexposed individuals only), whichever came first.

### Exposure variables

Bereavement was defined as the death of a family member (child, spouse, sibling, or parent), identified using data from the TPR, MGR, and Cause of Death Register (CDR). The CDR includes information on all dates and causes of deaths [28]. In addition to studying any bereavement, we also categorized exposure by the study participants’ relationship to the deceased, and by the specific underlying cause of death, including suicide, accident, COVID-19 (only relevant for study period 2), and other causes (Table A in S1 Appendix).

### Outcomes

Mental illness as a composite outcome was defined as either an incident diagnosis of psychiatric disorder or a first event of suicidal behavior during the study period. Incident mental illness was defined as the first hospital visit (inpatient or outpatient) where a psychiatric disorder was indicated as the primary or a secondary discharge diagnosis, according to the Swedish National Patient Register (NPR) (Table B in S1 Appendix); we included diagnoses of anxiety, depression, stress-related disorders and substance use disorder. The NPR collects information on inpatient (since 1964) and outpatient (since 2001) care for psychiatric disorders in Sweden, including dates of admission and discharge, main and secondary diagnoses made at discharge, etc [29]. Suicidal behavior was also included to indicate severe forms of mental illness, encompassing both suicide attempt and completed suicide, identified from the NPR and CDR, respectively (Table B in S1 Appendix). Apart from the primary composite outcome, we also studied the different types of psychiatric disorders and suicidal behavior separately, as secondary outcomes.

### Covariates

In addition to the matching factors (i.e., birth year, sex, and calendar time), we also included highest attained education and household income measured in 2017 (for study period 1) and 2019 (for study period 2) as two time-fixed covariates. Previous bereavement within 2 years prior to the index date was also included as a binary covariate, and COVID-19 status as a time-varying covariate (study period 2 only).

Data on education and household income was obtained from mandatory administrative records in the Swedish Longitudinal Integration Database for Health Insurance and Labour Market Studies (LISA) [30]. Unlike self-reported scales, these variables are based on objective registry classifications. Educational attainment was categorized according to the Swedish National Classification of Education (SUN). For individuals aged over 18 years, level of education was defined as primary education (≤9 years), upper secondary education (>9–12 years), and college/university (>12 years). For individuals under 18 years, this categorization was based on the highest educational level of either parent. Income level was categorized according to quartiles of the population distribution of the household disposable income per consumption unit (variable DISPINKKE04) [30]. COVID-19 status was classified as mild (positive test or outpatient hospital care), or severe (inpatient hospital care or ICU admission) using data from three national registers: 1) NPR; 2) the SmiNet (National COVID-19 notifiable disease reporting system) - Sweden#39;s mandatory infectious disease reporting system where healthcare providers report notifiable disease [31]; and 3) the Swedish Intensive Care Register (SIR), which monitors daily COVID-19 ICU admissions [32].

### Statistical analysis

For both study periods, we calculated the mean crude incidence rate (IR) of any first diagnosis of mental illness by dividing the number of incident cases by accumulated person-time during follow-up. To visualize temporal trends, we also estimated the cumulative incidence of any mental illness, by type of bereavement separately for study periods 1 and 2. To explore the potential impact of age, we estimated the cumulative incidence of mental illness by type of bereavement among different age groups.

Multivariable Cox regression models were run to estimate hazard ratios (HR) and 95% confidence intervals (CI), using time since the index date as the underlying time scale. The proportional hazards assumption was tested using scaled Schoenfeld residuals; we did not detect any major deviation. We performed a total of eighteen models for the primary outcome (i.e., risk of any mental illness) and the specific sub-outcomes (i.e., any psychiatric diagnosis, depression, anxiety, stress-related disorders, substance use disorders, any suicidal behavior, suicide attempt and death due to suicide), all of which were stratified by matching factors and adjusted for education, household income, prior bereavement history, and COVID-19 status for period 2. Stratified analyses were conducted by age, sex, education, household income, and COVID-19 status (study period 2 only). Separate analyses were also performed to examine whether the results varied by type of bereavement (i.e., loss of child, spouse, sibling, or parent) among different sex and age groups, as well as the cause of death of the deceased. For study period 2, we also examined death due to COVID-19. In addition to Cox models, we used multivariable flexible parametric models to visualize HR and 95% CI of any mental illness by time since bereavement for different age groups. We used Z-test to compare the IRs and HRs between study periods and participant groups.

As study participants could have experienced more than one loss during the study period, we conducted a sensitivity analysis in which individuals who experienced more than one loss during follow-up were censored at the time of the second event. To account for potential non-linear relationships between age at index date and the risk of mental illness, we further modeled age as a continuous variable, using restricted cubic splines [33].

Analyses were conducted using SAS version 9.4 and R version 4.4.1.

## Results

A total of 3,840,845 individuals (349,168 bereaved) were included in the pre-pandemic period and 5,132,988 individuals (466,636 bereaved) were included in study during the pandemic (Table 1). In both study periods, the bereaved and matched non-bereaved individuals had similar age and sex distributions; however, the bereaved individuals had slightly lower education compared to the matched non-bereaved group.

**Table 1:** Baseline characteristics before and during the COVID-19 pandemic for bereaved and their individually matched non-bereaved individuals – nationwide matched cohort studies during 2018-2021 in Sweden.

Characteristics	Pre-pandemic period (January 1^st^ 2018-December 31^st^ 2019) ^*^		Pandemic period (January 1^st^ 2020-December 31^st^ 2021) ^*^	
	Non-bereaved^†^	Bereaved^†^	Non-bereaved^†^	Bereaved^†^
Total N	3491677	349168	4666352	466636
Sex = Male, N (%)	1802777 (51.6)	180278 (51.6)	2243132 (48.1)	224313 (48.1)
Age group, N (%)				
10-17 years	24140 (0.7)	2414 (0.7)	27652 (0.6)	2765 (0.6)
18–29 years	130340 (3.7)	13034 (3.7)	144920 (3.1)	14492 (3.1)
30–44 years	438350 (12.6)	43835 (12.6)	524570 (11.2)	52457 (11.2)
45–59 years	1283990 (36.8)	128399 (36.8)	1581420 (33.9)	158142 (33.9)
60–74 years	1190690 (34.1)	119069 (34.1)	1550060 (33.2)	155006 (33.2)
75 + years	424167 (12.1)	42417 (12.1)	837730 (18.0)	83774 (18.0)
Highest education, N (%) ^‡^				
Primary school (≤9 years)	638086 (18.3)	65579 (18.8)	874492 (18.7)	90319 (19.4)
High school (>9–12 years)	1584794 (45.4)	164258 (47.0)	2091329 (44.8)	217264 (46.6)
College/university (>12 years)	1268797 (36.3)	119331 (34.2)	1700529 (36.4)	159053 (34.1)
Household income, N (%)				
Low	879414 (25.2)	81135 (23.2)	1177577 (25.2)	106335 (22.8)
Medium	1741239 (49.9)	178953 (51.3)	2324570 (49.8)	241293 (51.7)
High	871023 (24.9)	89080 (25.5)	1164201 (24.9)	119008 (25.5)
COVID-19 status, N (%) ^§^				
No registered COVID-19	–	–	4138912 (88.7)	411769 (88.3)
Mild	–	–	484853 (10.4)	50074 (10.7)
Severe	–	–	42587 (0.9)	4793 (1.0)

* Pre-pandemic period was defined as between January 1^st^, 2018, and December 31^st^, 2019; Pandemic period was defined as between January 1^st^, 2020, and December 31^st^, 2021.

† Up to 10 non-bereaved individuals who had not loss any family member between the start of study period and the index date were matched to each bereaved individual.

‡ For individuals at <18 years, highest education was defined by the highest education of the two parents.

§ A positive test of SARS-CoV-2 or an outpatient diagnosis for COVID-19 was classified as mild COVID-19, while inpatient care or ICU admission for COVID-19 was classified as severe COVID-19.

Bereaved individuals consistently demonstrated higher incidence rates (IR) of mental illness diagnoses than their non-bereaved counterparts in both study periods. During the pre-pandemic period (study period 1), the IR was 9.5 per 1000 person-years (95% CI 9.2-9.8) in bereaved individuals versus 6.7 (95% CI 6.6-6.8) in non-bereaved individuals. This pattern persisted during the pandemic period (study period 2), with IRs of 11.8 (95% CI 11.5-12.1) and 8.8 (95% CI 8.7-8.9) for bereaved and non-bereaved groups, respectively. Pairwise Z-tests indicated that IRs were consistently higher among bereaved individuals, compared to non-bereaved individuals, in both the pre-pandemic and pandemic periods (all p < 0.001). In addition, IRs increased from the pre-pandemic period to the pandemic period among both the bereaved and non-bereaved individuals (all p < 0.001) (Table C in S1 Appendix). Notably, the acute bereavement period (first 10 days post-loss) showed peak IRs of 30.0 (95% CI 26.9-33.9) and 30.7 (95% CI 27.6-33.7) per 1000 person-years in periods 1 and 2, representing approximately 3-fold increases compared to baseline rates Table 2.

**Table 2:** Incidence rate (IR) and 95% confidence interval (CI) of any mental illness during follow-up of the bereaved and their individually matched non-bereaved individuals – nationwide matched cohort studies during 2018-2021 in Sweden.

	Pre-pandemic period (January 1^st^ 2018-December 31^st^ 2019) *				Pandemic period (January 1^st^ 2020-December 31^st^ 2021) *			
	Non-bereaved		Bereaved		Non-bereaved		Bereaved	
Days since loss	No. of cases/person-yrs^†^	IR (95%CI), per 1000 person-yrs	No. of cases/person-yrs^†^	IR (95%CI), per 1000 person-yrs	No. of cases/person-yrs^†^	IR (95%CI), per 1000 person-yrs	No. of cases/person-yrs^†^	IR (95%CI), per 1000 person-yrs
Overall	23740/3535696	6.7 (6.6-6.8)	3456/363541	9.5 (9.2-9.8)	40430/4587409	8.8 (8.7-8.9)	5581/471926	11.8 (11.5-12.1)
0-10	627/94771	6.6 (6.1-7.1)	288/9478	30.0 (26.9-33.9)	1256/126403	9.9 (9.4-10.5)	388/12642	30.7 (27.6-33.7)
11-30	1081/185379	5.8 (5.5-6.2)	209/18567	11.3 (9.7-12.8)	2304/246801	9.3 (9-9.7)	381/24716	15.4 (13.9-17)
31-90	3373/523566	6.4 (6.2-6.7)	527/52664	10.0 (9.2-10.9)	6268/695481	9.0 (8.8-9.2)	879/69958	12.6 (11.7-13.4)
91-180	4673/699331	6.7 (6.5-6.9)	634/70922	8.9 (8.2-9.6)	8208/924700	8.9 (8.7-9.1)	1074/93836	11.4 (10.8-12.1)
181-365	7612/1120192	6.8 (6.6-6.9)	992/115384	8.6 (8.1-9.1)	13050/1472639	8.9 (8.7-9)	1678/151861	11.0 (10.5-11.6)
366-729	6593/912457	7.2 (7.1-7.4)	844/96526	8.7 (8.2-9.3)	9791/1121386	8.7 (8.6-8.9)	1251/118912	10.5(9.9-11.1)

* Pre-pandemic period was defined as between January 1^st^, 2018, and December 31^st^, 2019; Pandemic period was defined as between January 1^st^, 2020, and December 31^st^, 2021.

† Cases were defined as the first diagnosis of any psychiatric disorder of interest, or first event of suicide behavior including suicide attempt and completed suicide.

**Fig 3:**
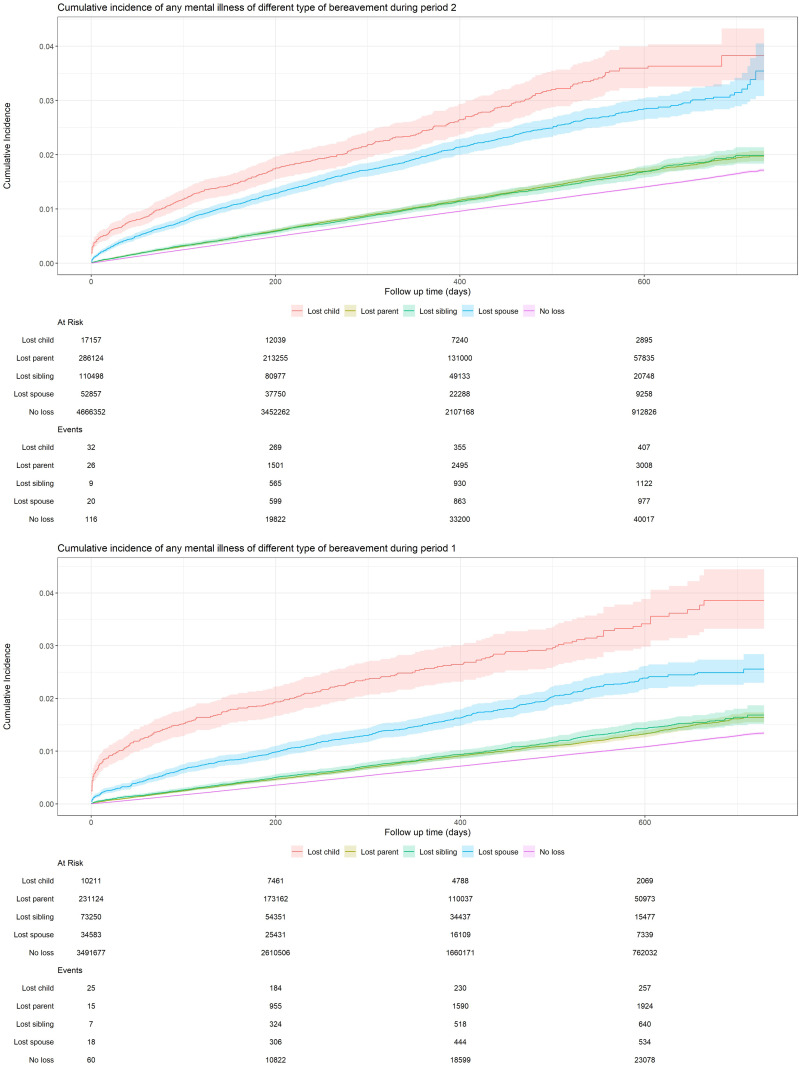
Cumulative incidence of psychiatric disorders or suicidal behavior in the pre-pandemic period (January 1^st^, 2018 – December 31^st^, 2019) and the pandemic period (January 1^st^, 2020 – December 31^st^, 2021).

In both study periods, individuals who lost a child or spouse had the highest cumulative incidence of any mental illness (Fig 3), except for the age group of 10–17 years for whom the greatest cumulative incidence was noted for loss of a parent (Fig A in S1 Appendix). In the age group of 45–59 years, individuals with loss of a child had the highest cumulative incidence, especially during study period 2. The confidence intervals for certain subgroups are relatively wide, reflecting limited statistical power due to small event counts in specific strata (Table D in S1 Appendix).

**Fig 4:**
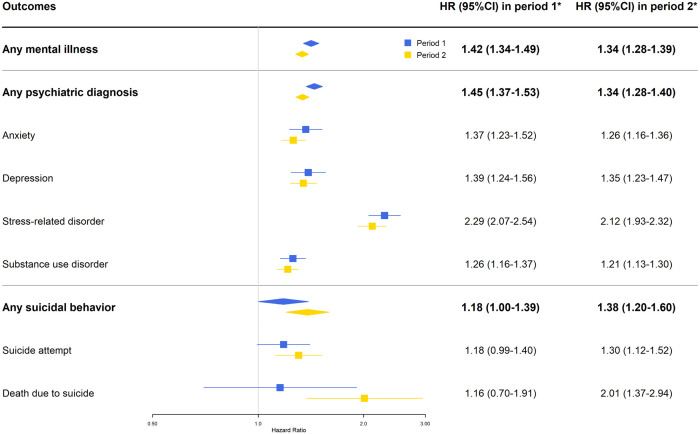
Risk of mental illness in relation to bereavement during the pre-pandemic period (January 1^st^, 2018 – December 31^st^, 2019) and the pandemic period (January 1^st^, 2020 – December 31^st^, 2021). * HRs and 95% CIs are presented on a logarithmic scale and adjusted for age, sex, calendar period, education, household income, prior bereavement for both periods, and COVID-19 status in the pandemic period. ‘Any mental illness’ was defined as the first diagnosis of any psychiatric disorder of interest, or first event of suicidal behavior. HR, hazard ratio; CI, confidence interval.

After multivariable adjustment, an increased risk of any mental illness was noted among the bereaved individuals, compared to the non-bereaved individuals, in both study periods (HR 1.42, 95% CI 1.34-1.49 in study period 1; HR 1.34, 95% CI 1.28-1.39 in study period 2) (Fig 4). This pattern was also observed for different types of psychiatric disorders as well as for suicide attempt and completed suicide, although the strongest association was observed for stress-related disorders (HR 2.29, 95% CI 2.07-2.54 in study period 1; HR 2.12, 95% CI 1.93-2.32 in study period 2).

**Fig 5:**
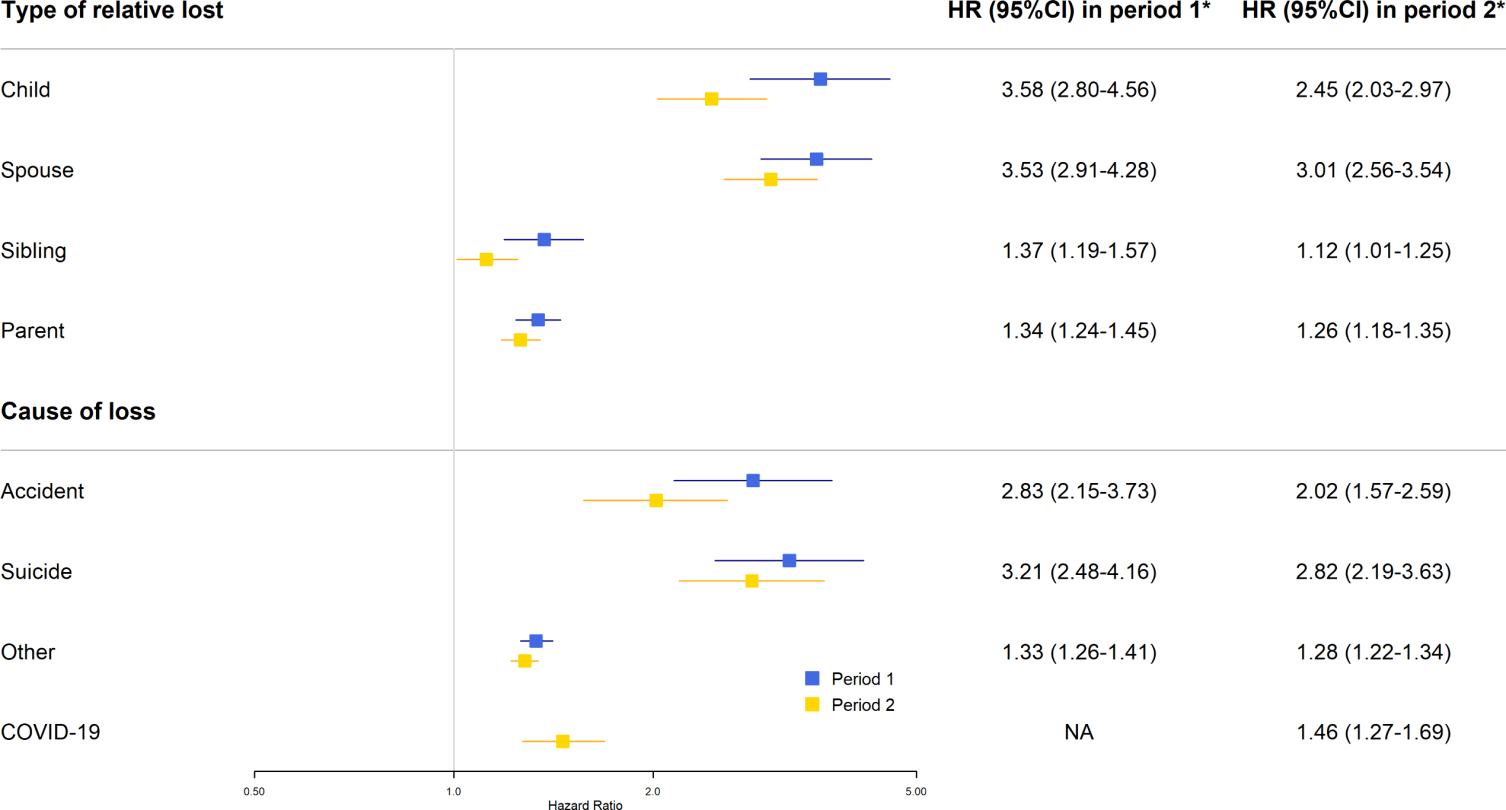
Risk of any mental illness in relation to bereavement during the pre-pandemic period (January 1st, 2018 – December 31st, 2019) and the pandemic period (January 1st, 2020 – December 31st, 2021) - stratified analysis by age, sex, education, household income, and COVID-19 status. * HRs and 95% CIs are presented on a logarithmic scale and adjusted for age, sex, calendar period, education, household income, prior bereavement for both periods, and COVID-19 status in the pandemic period. ‘Any mental illness’ was defined as the first diagnosis of any psychiatric disorder of interest, or first event of suicidal behavior. HR, hazard ratio; CI, confidence interval; NA, not applicable; Ref, reference group.

The stratified analyses showed higher HRs among children and adolescents (HR 2.52, 95% CI 1.84-3.44 in study period 1; HR 1.58, 95% CI 1.16-2.17 in study period 2), individuals with primary education only (HR 1.58, 95% CI 1.41-1.78 in study period 1; HR 1.45, 95% CI 1.31-1.59 in study period 2), and individuals with low household income (HR 1.58, 95% CI 1.44-1.74 in study period 1; HR 1.37, 95% CI 1.27-1.49 in study period 2) (Fig 5).

HRs of mental illness in relation to bereavement remained stable between the two periods, although some differences were observed among women and individuals with low household income between periods (Table E in S1 Appendix).

Flexible parametric models showed that the risk increment in mental illness was greatest immediately following bereavement (HR > 2.00 among all age groups) but progressively declined thereafter in both study periods (Fig B in S1 Appendix). However, bereaved individuals below 18 years exhibited a persistently elevated risk throughout the two-year follow-up period.

**Fig 6:**
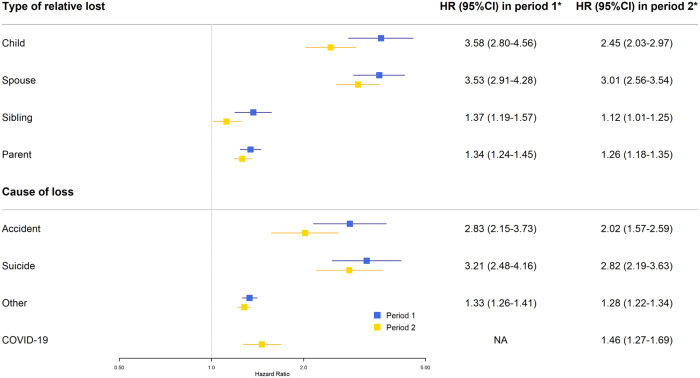
Risk of any mental illness in relation to bereavement during the pre-pandemic period (January 1st, 2018 – December 31st, 2019) and the pandemic period (January 1st, 2020 – December 31st, 2021) – analyses by the characteristics of bereavement. *HRs and 95% CIs are presented on a logarithmic scale and adjusted for age, sex, calendar period, education, household income, prior bereavement for both periods, and COVID-19 status in the pandemic period. ‘Any mental illness’ was defined as the first diagnosis of any psychiatric disorder of interest, or first event of suicidal behavior. The analysis by type of relative was restricted to individuals with the specific type of relatives alive before the start of follow-up during the respective study period. HR, hazard ratio; CI, confidence interval; NA, not applicable.

A greater risk increment in mental illness was observed for bereavement due to loss of a child (e.g., HR 3.58, 95% CI 2.80-4.56 in study period 1), or spouse (e.g., HR 3.01, 95% CI 2.56-3.54 in study period 2), compared to loss of a sibling or parent (Fig 6). Notably, among adolescents, the risk pattern shifted between periods: parental loss showed lower HRs during the pandemic period (HR 2.45, 95% CI 1.88-3.19 in study period 1; HR 1.42, 95% CI 1.08-1.87 in study period 2), whereas sibling loss demonstrated higher HRs during the pandemic period, compared to the pre-pandemic period (HR 1.47, 95% CI 0.63-3.44 in study period 1; HR 5.06, 95% CI 2.05-12.48 in study period 2) (Fig C in S1 Appendix). Although an increased risk of mental illness was noted in the bereaved individuals, regardless of the cause of death, the risk increment was greater for loss due to suicide (e.g., HR 2.82, 95% CI 2.19-3.63 in study period 2). In study period 2, loss due to COVID-19 was also associated with an increased risk of mental illness (HR 1.46, 95% CI 1.27-1.69).

Among the bereaved individuals, 2.72% and 2.90% had more than one bereavement in study period 1 and period 2, respectively. Results remained unchanged after censoring the follow-up of these individuals at the time of the second event (Fig D in S1 Appendix). The HR for mental illness was highest among the youngest individuals and generally attenuated with increased age. However, for loss of a child, a slightly greater HR was noted among the oldest individuals (Fig E in S1 Appendix).

## Discussion

We found that bereavement was consistently associated with increased risks of a first diagnosis of mental illness both before and during the pandemic, also after bereavement due to COVID-19. Consistent elevations in incident mental illness were observed following bereavement across both pre-pandemic and pandemic periods, with acute-phase vulnerability followed by gradual attenuation.

Contrary to our working hypothesis, the associations between bereavement and risk of mental illness were similar between the study periods, i.e., the COVID-19 pandemic did not seem to amplify the mental health impact of bereavement. One explanation to the limited differences in results between the pre-pandemic and pandemic periods may be Sweden’s comparatively non-mandatory COVID-19 mitigation strategy. Unlike the strict lockdowns implemented in many other countries, the Swedish approach relied primarily on voluntary physical distancing. Despite the enforcement of strict limits on attendance and gatherings, the Swedish approach permitted key mourning rituals to continue and social support networks to remain more accessible [34], which may have buffered against psychological distress [35]. Second, the reduced access to mental health services during the pandemic may have led to delays or missed diagnoses of mental illness in the general population and thereby influenced the observed associations [36]. Fig 4 supports this interpretation as patterns of suicidal behavior changed between the pre-pandemic and pandemic periods, and such outcomes are less dependent on healthcare utilization than other psychiatric diagnoses. At the same time, population-level data suggest that mental healthcare utilization (e.g., specialist visits, psychotropic medication use) and suicide rates remained stable in Sweden during 2018–2023, indicating overall resilience of the Swedish population [37].

As previously described, the associations observed between bereavement and risk of incident mental illness were similar across the study periods. However, given the higher absolute risk of mental illness among both the bereaved and non-bereaved individuals during the pandemic period, compared to the pre-pandemic period, bereavement was associated with a greater burden of mental illness on the absolute scale during the pandemic.

To our knowledge, this is the first large-scale population-based study in Sweden to compare mental health impact of bereavement between the pre-pandemic period and the pandemic period, with specific attention to: (1) COVID-19-related versus other causes of death, and (2) different type of family memberships (e.g., child loss, parent loss). Leveraging population registries, we analyzed incident mental illness and suicidal behavior. Our findings extended prior evidence from a Dutch study [38], which focused on self-reported grief symptoms rather than clinically recognized mental illness. However, our findings need to be interpreted in the context of Sweden’s non-mandatory mitigation strategies and similar studies in populations exposed to stricter social restrictions and extended periods of lockdown are warranted.

In this study, we identified incident mental illnesses through in- or outpatient specialist care among individuals with no prior such diagnosis recorded. As a result, milder mental illnesses not attended by specialist care were not included. Follow-up studies should focus on the potential synergistic effect between the pandemic and bereavement on milder mental illness, for instance, mental illnesses attended by primary care only.

However, it is important to note that it is difficult to distinguish grief from symptoms of mental illness. The observed risk increase in mental illness after bereavement, especially immediately following bereavement, might be, at least partially, attributed to intense grief reactions. Such experiences could have been recorded as a sub-type of stress disorder in the NPR, using the ICD-10 sub-code for adjustment disorder (F43.2). Moreover, substance use may further complicate grief, often serving as a coping mechanism for acute distress rather than a primary psychiatric disorder [39]. While these cases may not represent distinct comorbidities, their recording as a specialist diagnosis signifies that the survivor’s distress reached a clinical threshold requiring specialist intervention.

We observed that bereavement due to suicide was consistently associated with the greatest risk increment of mental illness during and before the pandemic. Individuals bereaved by suicide faced unique and complex stressors - including feelings of guilt, rejection, shame, anger, and the effects of stigma and trauma [40], therefore facing increased risks of depression, anxiety, and suicidal behavior compared to non-bereaved ones [8,10,40–42]. Additionally, bereavement due to loss of a child was associated with a particularly high risk for mental illness, especially in the middle-aged group who lost relatively young children. This is consistent with prior research showing that child loss is one of the most distressing types of bereavement with long-term severe emotional and psychological consequences [43–46]. The clinical nature of COVID-19, often involving rapid respiratory failure and acute physical distress, may be inherently more traumatic than many other natural causes of death. At the same time, restrictions on hospital visits during the pandemic, particularly for patients with COVID-19, often limited physical contact and bedside presence at the end of life [19]. Such circumstances might have intensified the psychological impact of bereavement in specific contexts, which may explain the slightly higher risk observed among individuals bereaved due to COVID-19 compared with individuals bereaved due to other natural deaths.

Bereaved individuals with a lower level of education and household income were found to have a significantly higher risk of mental illness, compared to individuals with a higher level, in the present study. This aligns with existing evidence showing socioeconomic factors as key determinants of mental health related health disparities, as financial strain and lack of resources may exacerbate psychological distress following a loss [47–49]. Population-based evidence from Sweden further suggests that socioeconomic disparities in mental health outcomes persisted during the COVID-19 pandemic, namely that individuals with lower socioeconomic status experienced disproportionately higher risks of poor mental health, as well as reduced access to healthcare, potentially amplifying post-bereavement vulnerability [50].

Age-related differences in vulnerability to mental illness following bereavement were also prominent in the study. Adolescents exhibited the greatest risk increment of mental illness as well as slower decline in such increment over time, compared to older individuals, suggesting a greater and prolonged trajectory of vulnerability. This suggests that experiencing bereavement early in life can significantly increase the risk of subsequent mental illness, and that such effect often persists for decades [6,8,51]. Notably, we also observed a subtle difference of mental illness risk among adolescents who experienced sibling loss between the two periods. The HR of mental illness in relation to sibling loss was slightly higher during the pandemic period compared to the pre-pandemic period, which may reflect disrupted family and social support during the pandemic [52,53].

This study has several strengths. First, the use of comprehensive national register data provided high-quality and systematically collected data on bereavement, mental illness, and sociodemographic characteristics, minimizing selection and information biases. We examined the risk of mental illness across different periods after bereavement, highlighting the immediate aftermath of bereavement as a high-risk time window. Furthermore, this study examined a wide range of mental illnesses, including different psychiatric disorders and suicidal behavior, providing a comprehensive understanding of the mental health burden associated with bereavement.

However, several limitations should be acknowledged. First, the use of register data only identified mental illness attended and diagnosed in the specialized care setting (diagnosis of psychiatric disorders or suicide attempt) or completed suicide. Relatively mild mental illness cases, attended by primary care alone or not attended by healthcare at all, could not be identified in this study. Meanwhile, people might have avoided healthcare due to limited access (especially specialized care) or fear of infection during the pandemic [54]. Second, although we included several sociodemographic factors (age, sex, calendar time, education, household income, and COVID-19 status) as covariates in the study, residual confounding cannot be excluded. For example, factors such as lifestyle factors, size of social network, social support, and access to health care were not adjusted for [55–57]. Third, our exclusion of individuals with pre-existing mental health conditions means these findings may not fully represent population-level risks [58]. Finally, given the recency of the COVID-19 pandemic, we could not assess the longer-term risk of mental illness following bereavement, comparing the pandemic period with a post-pandemic period. Caution should be taken when generalizing these findings to other countries with different healthcare systems and burden as well as mitigating strategies for COVID-19.

Overall, our findings suggest that bereavement poses a substantial risk to mental health, regardless of whether it occurs during a pandemic. Older age and limited social networks may exacerbate the psychological impact of bereavement. Future work should investigate the interplay between older age, social isolation, and pandemics such as the COVID-19 pandemic in relation to the mental health impact of bereavement, in Sweden and elsewhere, to inform targeted preventive strategies for high-risk populations. Furthermore, research quantifying the broader socioeconomic burden of mental illness following bereavement, including hospitalizations and long-term disability, is essential to better inform policy and develop interventions to mitigate the psychological impact of bereavement. Finally, whether the association between bereavement and mental illness could be modified by other man-made or natural disastrous events like wars and natural disasters remains to be examined.

## Conclusion

Bereavement was associated with a consistently increased risk of mental illness; however, this association did not appear to differ between the COVID-19 pandemic and a pre-pandemic period in Sweden. Specifically, factors such as bereavement by suicide or the loss of a child or spouse still play a dominant role in the adverse consequences of bereavement. The risk of mental illness was the highest immediately after the loss across both periods, and high-risk groups included children and adolescents as well as individuals with lower socioeconomic status. Regardless, these results substantiate the mental health impact of bereavement and highlight the need for sustained psychological support and interventions to mitigate such impact.

## Supporting information

S1 Appendix Supplementary figures and tables with internal legends. (DOCX)

S1 File STROBE Checklist. (DOC)
