# Supplementary material for: Mental illness after bereavement before and during the COVID-19 pandemic in Sweden: A matched cohort study
Source: PLOS Ment Health. 2026 May 6;3(5):e0000565. doi: 10.1371/journal.pmen.0000565 (PMC13148689; doi:10.1371/journal.pmen.0000565)
Supplement: S1 Appendix — (DOCX) [file pmen.0000565.s001.docx]

**S1 Appendix**

**Mental illness after bereavement before and during the COVID-19 pandemic: a matched cohort study in Sweden**

Shiyu Li, MSc^1*^, Mary M Barker, PhD^1^, Huiqi Li, PhD^2^, Krisztina László, PhD^3,4^, Fen Yang, PhD^1^, Mikael Rostila, PhD^5,6,7^, Sandra Rogne, MSc^6,7^, Filip K Arnberg, PhD^8^, Maria Feychting, PhD^1^, Unnur A Valdimarsdóttir, PhD^1,9,10^, Fredrik Nyberg, PhD^2¶^, Fang Fang, PhD^1¶^

1. Institute of Environmental Medicine, Karolinska Institutet, Stockholm, Sweden
2. School of Public Health and Community Medicine, Institute of Medicine, Sahlgrenska Academy, University of Gothenburg, Gothenburg, Sweden
3. Department of Global Public Health, Karolinska Institutet, Stockholm, Sweden
4. Department of Public Health and Caring Sciences, Uppsala University, Uppsala, Sweden
5. Department of Public Health Sciences, Stockholm University, Stockholm, Sweden
6. Centre for Health Equity Studies (CHESS), Stockholm University/Karolinska Institutet, Stockholm, Sweden
7. Centre for Health Equity Studies, Stockholm University/Karolinska Institutet, Stockholm University, Stockholm, Sweden
8. Department of Medical Sciences, National Centre for Disaster Psychiatry, Uppsala University, Uppsala, Sweden
9. Centre of Public Health Sciences, Faculty of Medicine, University of Iceland, Reykjavik, Iceland
10. Department of Epidemiology, Harvard T H Chan School of Public Health, Boston, Massachusetts, United States of America

* Corresponding author

Shiyu Li

Institute of Environmental Medicine, Karolinska Institutet, Stockholm, Sweden, 171 65

Tel: +46 72 001 6582

Email: shiyu.li@ki.se

¶ These authors contributed equally to this work

Table of Contents

[Table A: Underlying causes of death and corresponding ICD-10 codes 3](#_Toc227225258)

[Table B: Psychiatric disorders and suicidal behaviors and corresponding ICD-10 codes. 3](#_Toc227225259)

[Table C. Incidence rates (IR) of mental illness by bereavement status and study period, with pairwise Z-tests 3](#_Toc227225260)

[Table D. Numbers of different types of family member lost during the two study periods and numbers of any mental illness events – analysis by age group 4](#_Toc227225261)

[Table E. Comparison of Hazard Ratios (HRs) between two study periods across different subgroups 5](#_Toc227225262)

[Fig A: Cumulative incidence of psychiatric disorders or suicidal behavior in the pre-pandemic period (January 1st, 2018 – December 31st, 2019) and the pandemic period (January 1st, 2020 – December 31st, 2021) by age group 6](#_Toc227225263)

[Fig B: Hazard ratio (HR) and 95% confidence interval (CI) of any mental illness in relation to bereavement by time since bereavement in the pre-pandemic period (January 1st, 2018 – December 31st, 2019) and the pandemic period (January 1st, 2020 – December 31st, 2021) – analysis by age group 7](#_Toc227225264)

[Fig C: Hazard ratio (HR) and 95% confidence interval (CI) of any mental illness in relation to different types of bereavement during the pre-pandemic period (January 1st, 2018 – December 31st, 2019) and the pandemic period (January 1st, 2020 – December 31st, 2021) - stratified analysis by sex and age 8](#_Toc227225265)

[Fig D: Hazard ratio (HR) and 95% confidence interval (CI) of any mental illness in relation to different kinds of bereavement during the pre-pandemic period (January 1^st^, 2018 – December 31^st^, 2019) and the pandemic period (January 1^st^, 2020 – December 31^st^, 2021) - sensitivity analysis with censoring at the time of a second bereavement event 9](#_Toc227225266)

[Fig E: Age-specific hazard ratios of any mental illness in relation to bereavement by time since bereavement in the pre-pandemic period (January 1st, 2018 – December 31st, 2019) and the pandemic period (January 1st, 2020 – December 31st, 2021) 10](#_Toc227225267)

# **Table A: Underlying causes of death and corresponding ICD-10 codes**

| **Causes** | **ICD-10 (1997-)** |
| --- | --- |
| All causes | A00-Y89 |
| COVID-19 related | U07.1, U07.2, U09.9, U10.9 |
| Suicidal | X60-X84, Y10-Y34 |
| Accidental | V01-X59 |

# **Table B: Psychiatric disorders and suicidal behaviors and corresponding ICD-10 codes.**

| Psychiatric disorders or suicidal behaviors | | ICD-10 (1997-) |
| --- | --- | --- |
| Any mental illness | | F10-F69, X60-X84, Y10-Y34 |
| Any psychiatric disorder | | F10-F69 |
| Substance use disorder | F10-F19 |  |
| Depression | F32-F33 |  |
| Anxiety | F40-F41 |  |
| Stress-related disorders | F43 |  |
| Suicide attempt and completed suicide | | X60-X84, Y10-Y34 |
| Intentional self-harm | X60-X84 |  |
| Injury/poisoning of undetermined intent | | Y10-Y34 |

# **Table C. Incidence rates (IR) of mental illness by bereavement status and study period, with pairwise Z-tests**

|  | **Pre-pandemic period (January 1st 2018-December 31st 2019)**  **(IR per 1,000 person-years, 95% confidence interval)** | **Pandemic period (January 1st 2020-December 31st 2021)**  **(IR per 1,000 person-years, 95% confidence interval)** | **IR difference** | **Z value** | **P value** |
| --- | --- | --- | --- | --- | --- |
| Non-bereaved | 6.7 (6.6-6.8) | 8.8 (8.7-8.9) | 2.1 | 29.2 | <0.001 |
| Bereaved | 9.5 (9.2-9.8) | 11.8 (11.5-12.1) | 2.3 | 10.6 | <0.001 |
| IR difference | 2.8 | 3.0 | - | - | - |
| Z value | 17.4 | 18.6 | - | - | - |
| P value | <0.001 | <0.001 | - | - | - |

# **Table D. Numbers of different types of family member lost during the two study periods and numbers of any mental illness events – analysis by age group**

|  | **Period 1 (before pandemic) *** | | | | | **Period 2 (during pandemic) *** | | | |
| --- | --- | --- | --- | --- | --- | --- | --- | --- | --- |
| **Numbers of individuals** | **Child** | **Spouse** | **Sibling** | **Parent** | **Child** | | **Spouse** | **Sibling** | **Parent** |
| Age group |  |  |  |  |  | |  |  |  |
| 10-17 years | 3 | - | 273 | 2138 | - | | - | 309 | 2456 |
| 18-29 years | 225 | 67 | 1206 | 11536 | 286 | | 84 | 1255 | 12867 |
| 30-44 years | 737 | 777 | 2307 | 40014 | 867 | | 928 | 2607 | 48055 |
| 45-59 years | 1751 | 2947 | 10615 | 113086 | 2169 | | 4070 | 12524 | 139379 |
| 60-74 years | 3208 | 13305 | 39385 | 63171 | 4261 | | 17430 | 52058 | 81257 |
| 75+ years | 4287 | 17487 | 19464^¶^ | 1179^¶^ | 9574 | | 30345 | 41745^¶^ | 2110^¶^ |
| Total | 10211 | 34583 | 73250 | 231124 | 17157 | | 52857 | 110498 | 286124 |
| **Numbers of mental illness events** | **Child** | **Spouse** | **Sibling** | **Parent** | **Child** | | **Spouse** | **Sibling** | **Parent** |
| Age group |  |  |  |  |  | |  |  |  |
| 10-17 years | - | - | 5 | 83 | - | | - | 14 | 76 |
| 18-29 years | 21 | 5 | 37 | 226 | 26 | | 2 | 35 | 282 |
| 30-44 years | 50 | 26 | 41 | 384 | 43 | | 35 | 43 | 581 |
| 45-59 years | 67 | 59 | 90 | 803 | 113 | | 89 | 155 | 1300 |
| 60-74 years | 56 | 210 | 417 | 328 | 92 | | 384 | 508 | 746 |
| 75+ years | 63 | 234 | 139 | 112 | 133 | | 503 | 367 | 23 |
| Total |  |  |  |  |  | |  |  |  |
| * Study period 1 was defined as between January 1, 2018, and December 31, 2019; Study period 2 was defined as between January 1, 2020, and December 31, 2021.  ^¶^ In the Swedish Multi-Generation Register, parental information is primarily available for individuals born in 1932 or later (and who were alive in 1947). For the oldest participants (75+), the system often lacks the PINs of their parents, which consequently limits our ability to identify their siblings. | | | | | | | | | |

# **Table E. Comparison of Hazard Ratios (HRs) between two study periods across different subgroups**

|  | **Pre-pandemic period *** | **Pandemic period *** | **Z value** | **P value** |
| --- | --- | --- | --- | --- |
| **Outcomes** | | | | |
| Any mental illness | 1.42 (1.34-1.49) | 1.34 (1.28-1.39) | 1.69 | 0.090 |
| Any psychiatric diagnosis | 1.45 (1.37-1.53) | 1.34 (1.28-1.40) | 2.17 | 0.029 |
| Anxiety | 1.37 (1.23-1.52) | 1.26 (1.16-1.36) | 1.23 | 0.215 |
| Depression | 1.39 (1.24-1.56) | 1.35 (1.23-1.47) | 0.39 | 0.693 |
| Stress-related disorder | 2.29 (2.07-2.54) | 2.12 (1.93-2.32) | 1.09 | 0.271 |
| Substance use disorder | 1.26 (1.16-1.37) | 1.21 (1.13-1.30) | 0.70 | 0.465 |
| Any suicide behavior | 1.18 (1.00-1.39) | 1.38 (1.20-1.60) | -1.40 | 0.160 |
| Suicide attempt | 1.18 (0.99-1.40) | 1.30 (1.12-1.52) | -0.82 | 0.411 |
| Completed suicide | 1.16 (0.70-1.91) | 2.01 (1.37-2.94) | -1.70 | 0.087 |
| **Age group** | | | | |
| 10-17 years | 2.52 (1.84-3.44) | 1.58 (1.16-2.17) | 1.84 | 0.065 |
| 18-29 years | 2.02 (1.69-2.43) | 1.62 (1.36-1.95) | 1.82 | 0.068 |
| 30-44 years | 1.67 (1.46-1.91) | 1.41 (1.25-1.58) | 1.80 | 0.071 |
| 45-59 years | 1.19 (1.09-1.31) | 1.27 (1.17-1.37) | -1.14 | 0.251 |
| 60-74 years | 1.31 (1.19-1.44) | 1.31 (1.21-1.42) | 0.00 | 1.000 |
| 75+ years | 1.56 (1.35-1.79) | 1.34 (1.22-1.47) | 1.77 | 0.075 |
| **Sex** | | | | |
| Female | 1.52 (1.41-1.63) | 1.34 (1.27-1.42) | 2.38 | 0.017 |
| Male | 1.32 (1.23-1.42) | 1.33 (1.25-1.42) | -0.15 | 0.876 |
| **Highest education level** | | | | |
| Primary school | 1.58 (1.41-1.78) | 1.45 (1.31-1.59) | 1.12 | 0.258 |
| High school | 1.37 (1.28-1.47) | 1.32 (1.24-1.40) | 0.82 | 0.409 |
| College/university | 1.39 (1.25-1.53) | 1.29 (1.19-1.40) | 1.12 | 0.262 |
| **Household income level** | | | | |
| Low | 1.58 (1.44-1.74) | 1.37 (1.27-1.49) | 2.08 | 0.037 |
| Medium | 1.39 (1.30-1.49) | 1.33 (1.26-1.41) | 1.00 | 0.314 |
| High | 1.17 (1.01-1.36) | 1.27 (1.13-1.43) | -0.887 | 0.3753 |

**Fig A: Cumulative incidence of psychiatric disorders or suicidal behavior in the pre-pandemic period (January 1st, 2018 – December 31st, 2019) and the pandemic period (January 1st, 2020 – December 31st, 2021) by age group**


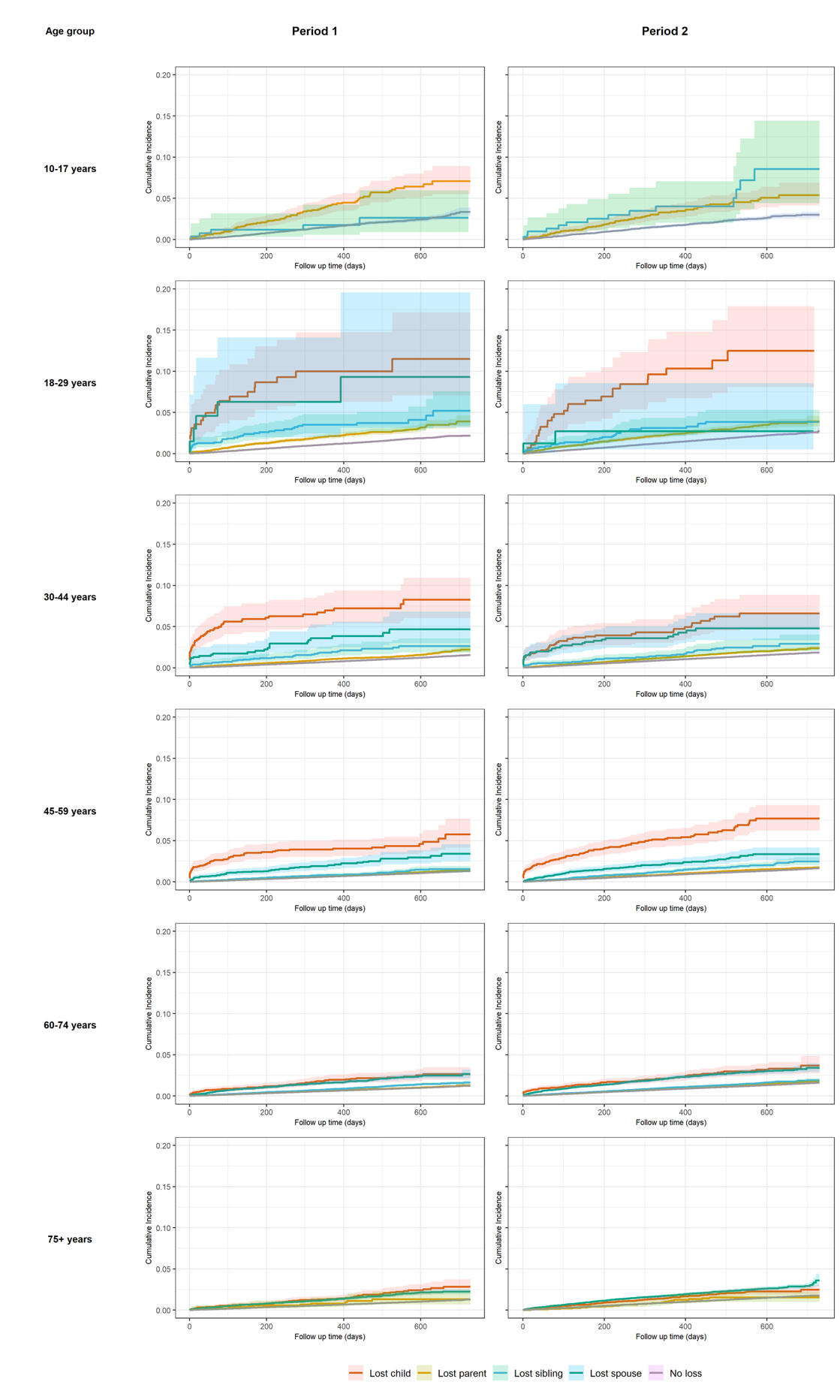


**Fig B: Hazard ratio (HR) and 95% confidence interval (CI) of any mental illness in relation to bereavement by time since bereavement in the pre-pandemic period (January 1st, 2018 – December 31st, 2019) and the pandemic period (January 1st, 2020 – December 31st, 2021) – analysis by age group**
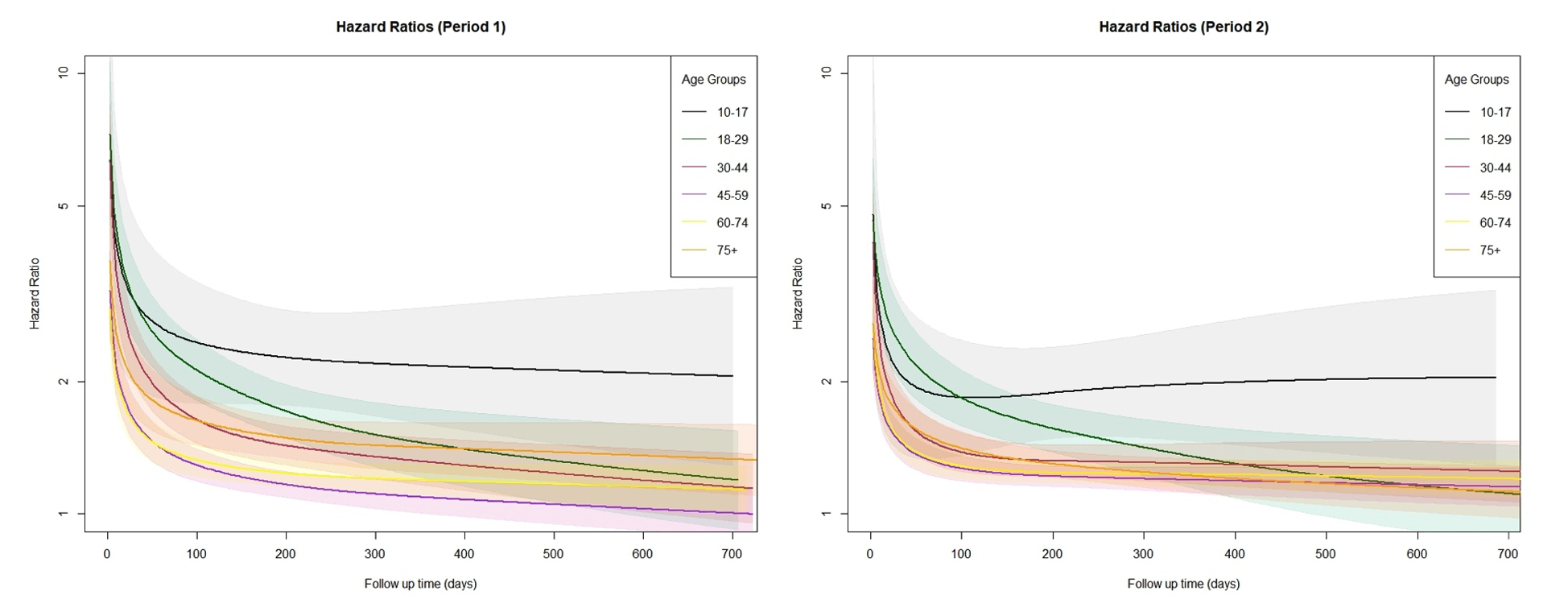


HRs and 95% CIs are derived from flexible parametric models and presented on a logarithmic scale, after adjustment for matching factors (age, sex and index ddate), education, household income, prior bereavement for both periods, and COVID-19 status for study period 2. ‘Any mental illness’ was defined as the first diagnosis of any psychiatric diagnosis of interest, or first event of any suicidal behavior. The solid curves represent HRs and the shaded areas are 95%CIs. HR, hazard ratio; CI, confidence interval.

# **Fig C: Hazard ratio (HR) and 95% confidence interval (CI) of any mental illness in relation to different types of bereavement during the pre-pandemic period (January 1st, 2018 – December 31st, 2019) and the pandemic period (January 1st, 2020 – December 31st, 2021) - stratified analysis by sex and age**


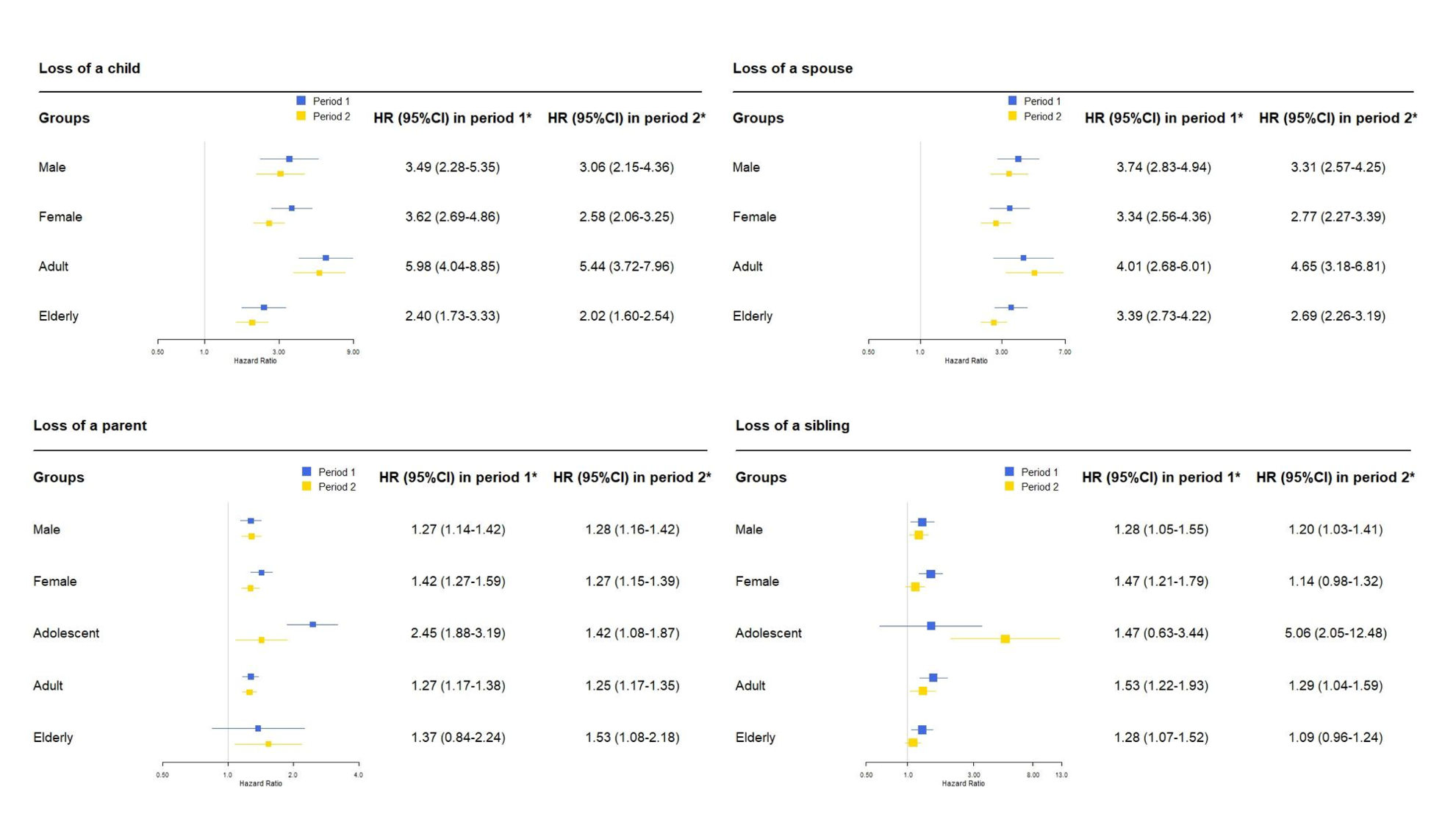


* HRs and 95%CIs are presented on a logarithmic scale, after adjustment for matching factors (age, sex, and the index date), education, household income, prior bereavement for both periods, and COVID-19 status for study period 2. ‘Any mental illness’ was defined as the first diagnosis of any psychiatric disorder of interest, or first event of any suicidal behavior. HR, hazard ratio; CI, confidence interval. Adolescent - 10-17 years; adult - 18-64 years; elderly - over 65 years

**Fig D: Hazard ratio (HR) and 95% confidence interval (CI) of any mental illness in relation to different kinds of bereavement during the pre-pandemic period (January 1^st^, 2018 – December 31^st^, 2019) and the pandemic period (January 1^st^, 2020 – December 31^st^, 2021) - sensitivity analysis with censoring at the time of a second bereavement event**


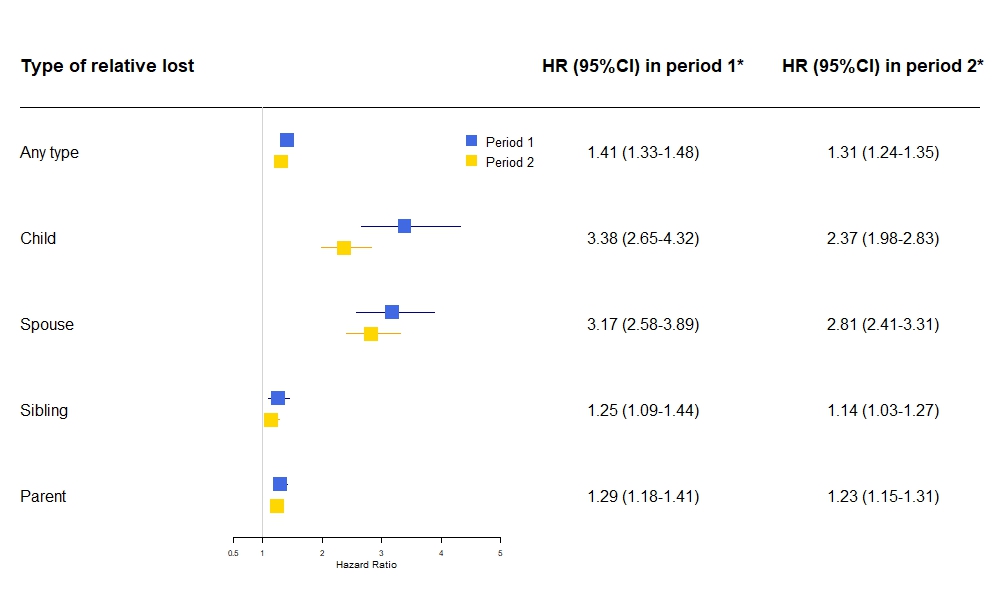


*HR and 95% CIs are presented on a logarithmic scale, after adjustment for matching factors (age, sex, and the index date), education, household income, prior bereavement for both periods, and COVID-19 status for study period 2. ‘Any mental illness’ was defined as the first diagnosis of any psychiatric disorder of interest, or first event of any suicidal behavior. The analysis of type of relative was restricted to individuals who had the relatives of interest alive on the index date during the respective study period. Loss of a second family member during the follow-up was considered as a censoring event. HR, hazard ratio; CI, confidence interval

**Fig E: Age-specific hazard ratios of any mental illness in relation to bereavement by time since bereavement in the pre-pandemic period (January 1st, 2018 – December 31st, 2019) and the pandemic period (January 1st, 2020 – December 31st, 2021)**


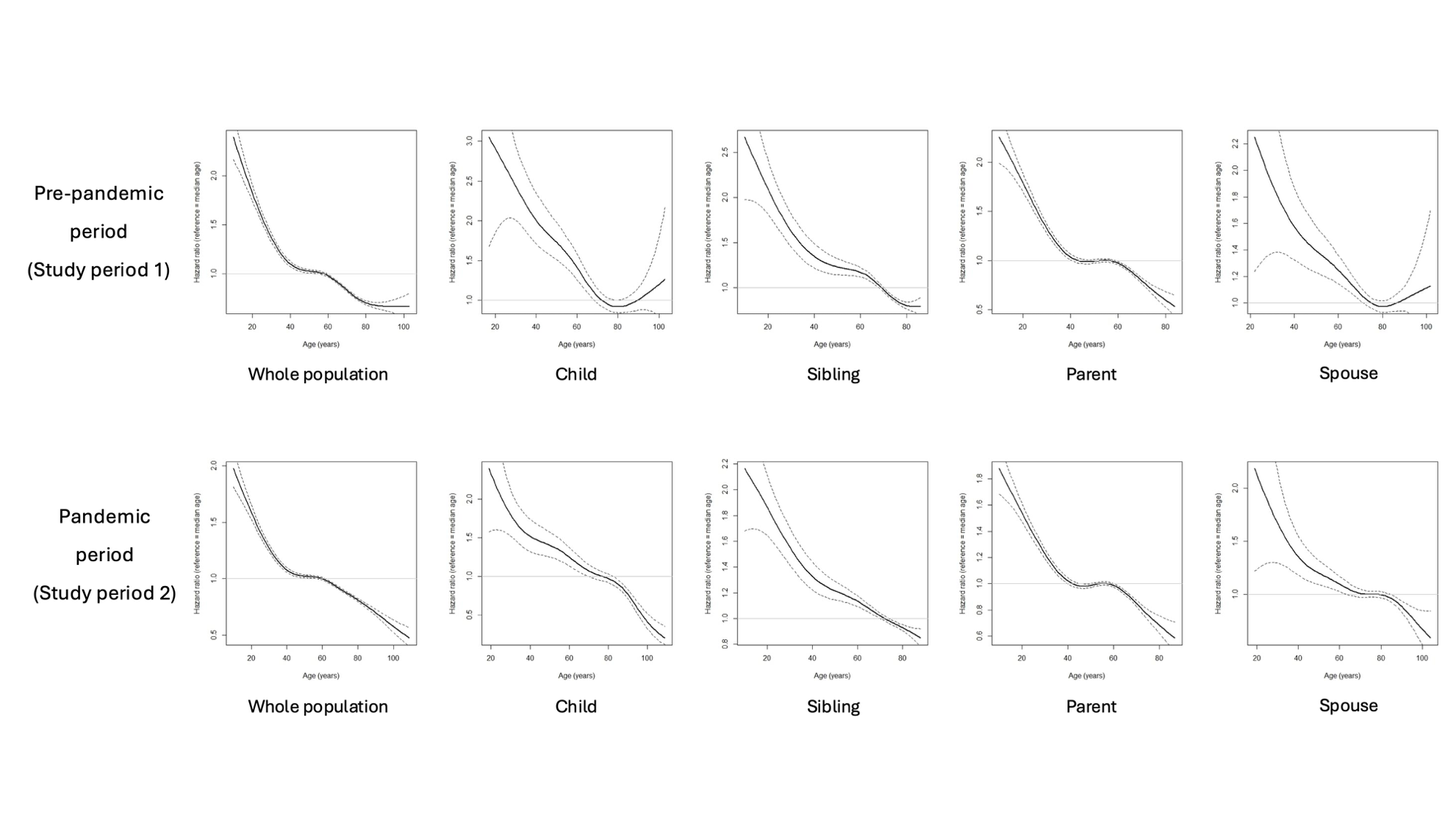


Hazard ratios (HRs) and 95% confidence intervals for the risk of incident mental illness following the loss of a child, parent, sibling, spouse, or any type, modeled as a function of age at bereavement after adjustment for sex, education, household income, prior bereavement for both periods, and COVID-19 status for study period 2. ‘Any mental illness’ was defined as the first diagnosis of any psychiatric diagnosis of interest, or first event of any suicidal behavior. The solid curves represent HRs and the dotted curves are 95%CIs. HR, hazard ratio; CI, confidence interval.
